# High-Throughput Cryopreservation of *In Vivo*-Derived Swine Embryos

**DOI:** 10.1371/journal.pone.0065545

**Published:** 2013-06-07

**Authors:** Lee D. Spate, Clifton N. Murphy, Randall S. Prather

**Affiliations:** Division of Animal Sciences, University of Missouri, Columbia, Missouri, United States of America; Michigan State University, United States of America

## Abstract

Cryopreservation of swine embryos is inefficient. Our goal was to develop a non-invasive method for “relatively” high-throughput cryopreservation of in vivo-produced swine embryos. Since removal of the lipid droplets within early swine embryos improves cryosurvival we wanted to apply a technique of high osmolality treatment followed by centrifugation that was first developed for in vitro-produced swine embryos to in vivo-produced swine embryos. The first aim was to determine how sensitive the in vivo-produced zygote and 2-cell stage embryo was to various high osmolality conditions for a short duration. Culture for 6, 12 or 18 min at 300, 400 or 500 milliosmoles (mOsm) had no detectable affect on the resulting blastocyst stage embryos (number of inner cell mass nuclei, trophectoderm nuclei, total number of nuclei, ratio of the trophectoderm to inner cell mass nuclei or percent blastocyst). However there was an effect of gilt on each of these parameters. For the second aim we focused on 300 mOsm for 6 min, 400 mOsm for 12 min, 500 mOsm for 12 min, and 500 mOsm for 18 min. The embryos were centrifuged for the duration of high osmolality treatment, then cultured to the blastocyst stage and vitrified. After vitrification and thawing the 500 mOsm for 18 min had the highest percent re-expansion with no difference in the total number of nuclei. While requiring a different base culture medium than in vitro-produced embryos, in vivo-derived embryos also survive cryopreservation without damage to their zona pellucida.

## Introduction

Cryopreservation of early mammalian embryos provides prospects for the preservation of germplasm as well as the movement of genetics nationally and internationally. Unfortunately the swine embryo has been more resistant to cryopreservation than the embryos of many mammals. The first significant advance toward the successful cryopreservation of swine embryos was based on the observation that they are very sensitive to hypothermic conditions and that removal of intracellular lipids (delipation) alleviates this sensitivity [1]–[4]. Alternatives for physically removing the lipids at an early stage include culturing to the blastocyst stage and then removing the lipids [5], destabilizing the cytoskeleton [6], altering the vitrification conditions [7]–[9], or chemically removing the lipids before cryopreservation [10]. While the chemical lipid removal appears attractive, there have yet to be any offspring produced by this method. Other cumbersome (slow-cooling) cryopreservation methods have been developed, but there is tremendous batch to batch variation [11].

The current methods for cryopreservation of swine embryos are time consuming and troublesome. One of the most successful methods of cryopreservation of swine embryos is to centrifuge the cells to polarize the lipids and then use a micromanipulator to remove the lipids from the embryo [4], [12]. Interestingly, removal of the lipids not only permits the embryos to survive low temperatures [13], but in some cases they actually develop to the blastocyst stage at a higher rate [14], [15]. Unfortunately this current procedure results in a break in the zona pellucida. The International Embryo Transfer Society guidelines state that the zona pellucida cannot be compromised if the health status is to be maintained [16], [17]. This is especially important for embryos that are to be frozen and transported to another facility, as swine are generally raised in specific pathogen-free environments and the movement of pathogens can have a devastating effect on the recipient herd.

While hyperosmotic treatments and centrifugation have been developed for lipid removal that does not damage the zona pellucida, these techniques have been only reported for in vitro-produced (IVP) embryos [18]. The limited information available on IVP swine oocytes suggests that they are very sensitive to osmotic stresses [19]. These authors showed that any deviation away from 290 milliosmolal (mOsm) resulted in a dramatic reduction in development after fertilization. The IVP blastocyst stage embryo is less sensitive to osmotic stress. When sucrose was used adjust the osmolality as long as the osmolality did not go beyond 170% of isotonic, i.e. ∼600 mOsm, then the embryos survived the osmotic insult [20].

Our goal was to develop a non-invasive method for “relatively” high-throughput cryopreservation of in vivo-produced swine embryos. Our first aim was to confirm that the amount of perivitteline space in the zona pellucida can be increased so that more complete separation of the lipids and the zygote cytoplasm can be achieved by centrifugation. The first aim was to determine how sensitive the in vivo-produced embryo is to various high osmolality conditions for a short duration. The second aim was to use the added perivitteline space, caused by high osmolality treatment, to aid in separating the lipids within the zona pellucida (via centrifugation) prior to cryopreservation and to determine their viability.

## Results

The experimental treatments for the first aim were designed to determine if high osmolality for a short duration will negatively affect development of zygotes and 2-cell stage embryos to the blastocyst stage. The specific treatments were: 1. 6 min at 300 mOsm, 2. 6 min at 400 mOsm, 3. 6 min at 500 mOsm, 4. 12 min at 400 mOsm, 5. 18 min at 400 mOsm, 6. 12 min at 500 mOsm, and 7. 18 min at 500 mOsm. These treatments were chosen based on the successful treatment of IVP embryos. As predicted we observed no treatment effects (p>0.15) on the number of inner cell mass (ICM) nuclei, trophectoderm (TE) nuclei, total number of nuclei, the ratio of the number of TE/ICM nuclei, or the percent blastocyst (Table 1). The power of the test ranged from 0.16 to 0.77. However, it was quite interesting to observe an effect due to gilt on every parameter (Table 1). While the power is not extremely high, we did have enough confidence in our results to move forward with the more important test of the ability of these embryos to survive the centrifugation and cryopreservation. In conclusion we found that there was no negative effect of osmotic stress on development of the embryos.

**Table 1:** In vitro development of in vivo-derived swine embryos after hyperosmotic treatment and centrifugation (the number of gilts providing embryos for this experiment was 21).

	Mean ± SEM (N)	Treatment Effect (p Value)	Power of Test	Gilt Effect (p Value)
# ICM Nuclei	7.83±0.38 (45)	0.60	0.35	<0.00
# TE Nuclei	23.20±1.00 45)	0.36	0.77	0.01
Total # Nuclei	32.89±0.87 (113)	0.15	0.37	0.01
Ratio TE/ICM	3.28±0.21 (45)	0.94	0.16	0.06
Percent Blastocyst	73.0±8.1 (135)	0.50	0.33	<0.01

**Figure 1:**
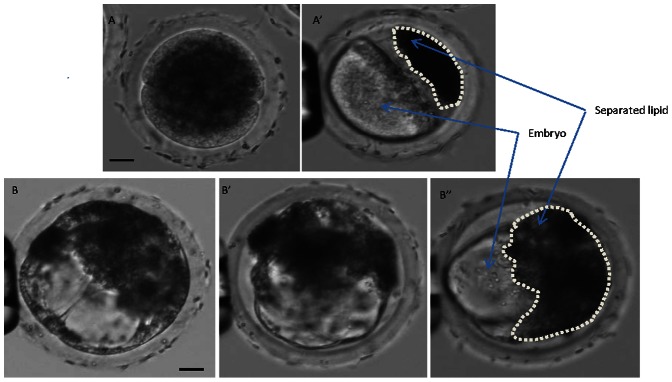
Separation of lipids in early pig embryos by centrifugation and hypertonicity. Two views of the same 2-cell stage embryo (A, A’) after exposure to a hypertonic solution and centrifugation. A 2-cell stage embryo that was previously subjected to a hypertonic solution and centrifugation followed by culture to the blastocyst stage (B, B’, B’’). The same embryo is held at different angles. In panel B’ and B” the embryo has been placed into another hypertonic solution to shrink the cells so that the lipids can be better visualized. The white dashed line encircles the extruded lipids. Scale bar = 25 microns.

**Figure 2:**
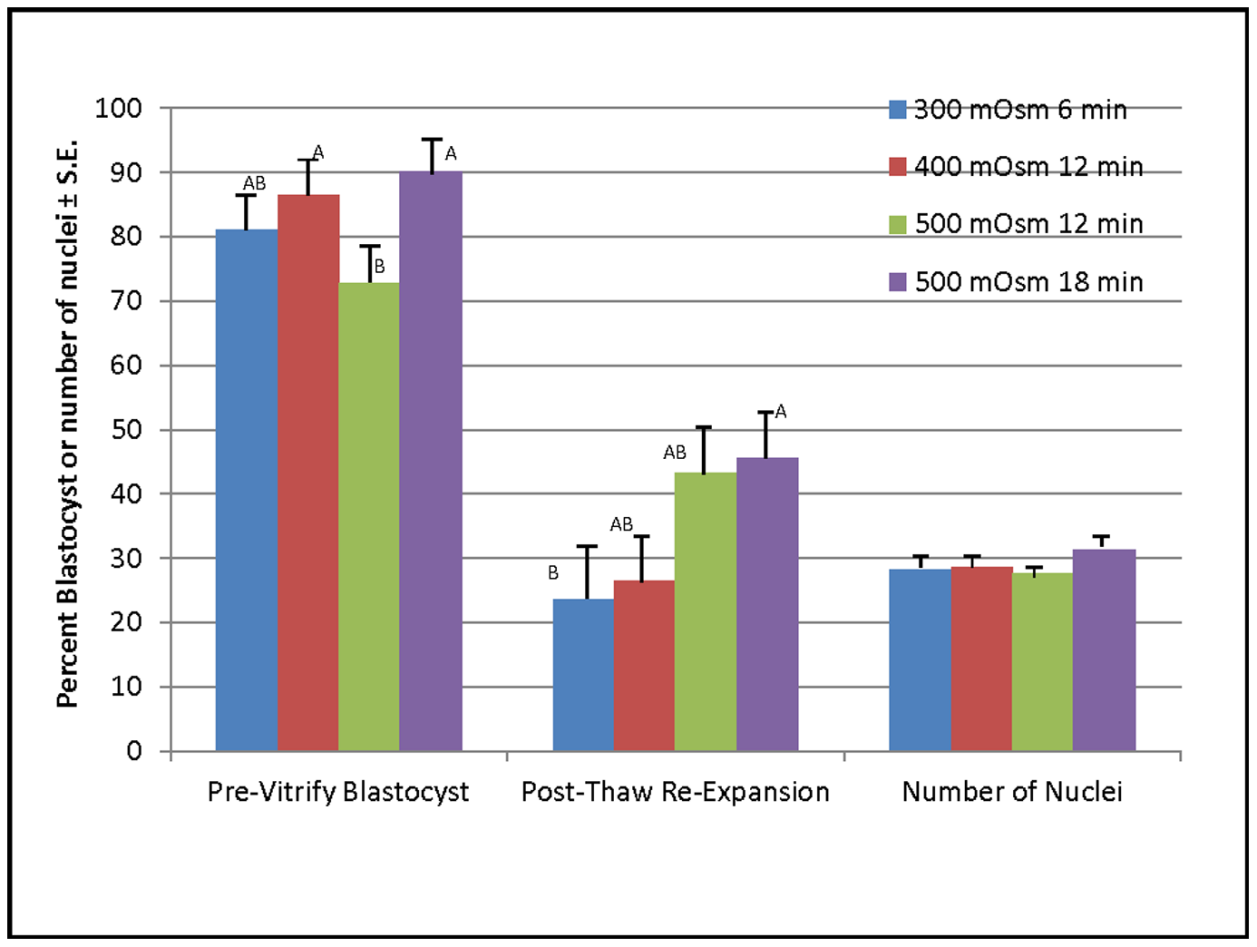
Blastocyst development and number of nuclei in embryos after various osmolality treatments. (^AB^P<0.05; N = 160 for blastocyst development, and 113 for number of nuclei; 23 different gilts provided embryos for this experiment.).

Experiments addressing the second aim were designed to reduce the number of treatment groups to maximize viability, and decrease the number of embryos that we would need to complete the project. Based on the previous data the treatments for this experiment were as follows: 1) 300 mOsm centrifuged for 6 min, 2) 400 mOsm centrifuged for 12 min, 3) 500 mOsm centrifuged for 12 min, or 4) 500 mOsm centrifuged for 18 min. Each of these treatments successfully disconnected the membrane-bound lipids from the remainder of the embryo (see Figure 1 for an example). It was expected that the ability to break this connection would be directly proportional to their subsequent development after cryopreservation. The percent of treated embryos that developed to the blastocyst stage was recorded. Blastocysts were then vitrified in open pulled straws (OPS) [18]. The embryos were later thawed in TCM 199 using a 2-step sucrose rehydration method [18]. The embryos were cultured for an additional 18 hours in PZM3 [21], assessed for blastocoel re-expansion and were processed to count total cell number. The percent blastocyst was higher in the 400 mOsm for 12 min and 500 mOsm for 18 min, than for 500 mOsm for 12 min (Fig. 2). After cryopreserving and thawing the embryos and culturing an additional 18 hours the re-expansion rates were higher from the 500 mOsm 18 min group as compared to the 300 mOsm group, but the total number of nuclei was not different (Fig. 2).

## Discussion

Swine oocytes and embryos have large, abundant lipid droplets in the early stage swine embryo and gradually decline in size and abundance as the embryo advances to and beyond the blastocyst stage [22], [23]. Coincident with reduced lipid content is an increased freezability of late stage embryos. Late stage embryos (hatched blastocyst) with low lipid content and smaller size of lipid droplets survive cryopreservation better than early stage embryos (2-cell, 4-cell and 8-cell) [24]–[26]. Interestingly, the large lipid droplets in the early stage embryos are easier to remove by centrifugation than the smaller droplets in the later stage embryos. While there is very little in the literature about the exact composition of the lipids in the swine oocyte; one study shows that 74 ng of fatty acids could be extracted from a single swine oocyte [27], and that this was significantly higher (3X) than even sheep and cattle oocytes (whose cytoplasm is also opaque).

Successful cryopreservation of IVP embryos has been reported after mechanical delipation through centrifugation and micromanipulation [14], [28]. However mechanical delipation substantially increases the potential of pathogen transmission because of the damage inflicted upon the zona pellucida during micromanipulation. It is also labor–intensive and time-consuming. Another option is to use hatched blastocysts as they are more cold tolerant than are zona enclosed blastocysts [29]. However the use of hatched blastocysts creates problems with maintaining the embryo’s barrier to diseases. International shipment of embryos requires that the zona pellucida remain intact. Development of a practical non-invasive means of lipid removal for cryopreservation of swine embryos is still necessary.

Removal of the lipids after centrifugation appears to be the most promising technique. However, after centrifugation of the swine oocyte or embryo with an intact zona pellucida the polarized lipid droplets remain connected with the cytoplasm of the oocyte or blastomere of the embryo via a bridge-like structure [13]. If the lipids are not immediately removed, or the embryo frozen, the polarized lipid droplets redistribute into the oocyte or blastomere during subsequent culture. Thus embryos need to be cryopreserved immediately after centrifugation in order to prevent lipid redistribution prior to cryopreservation [30]; however this is not practical since the lipid removal is best achieved during the early cleavage stages, and the embryos survive the best if they have developed to the blastocyst stage. If the perivitelline space is enlarged, the bridge-like structure may more easily break after centrifugation and the lipid droplets will not redistribute into the cytoplasm of the oocyte or the blastomere of the embryo, but will stay within the intact zona pellucida. At least two methods might make the perivitelline space larger. One is to swell the zona pellucida through partial enzymatic digestion (such as trypsin or pronase, etc). Another option is to condense the volume of the oocyte or embryo by high osmolality treatment as described here. Our treatment appeared to successfully break this connection between the embryo and the extruded lipids (Fig. 1). However based on this experiment alone, since it is difficult to visualize any lipids moving back to the embryo it cannot be concluded that both high osmolality and centrifugation are necessary for subsequent cryosurvival. But since centrifugation alone results in the lipids returning to the embryo and the embryo not surviving cryopreservation and since the high osmolality treatment occurs days before cryopreservation, it is likely that both are required for survival. Indeed, we propose that the higher centrifugation and hypertonic treatment work together to improve the lipid separation, and the higher cryosurvival is a direct indication of the success of lipid separation. Since this describes the development of a high throughput method of cryopreservation dozens of embryos can be treated at single time and thus avoids the necessity of micromanipulating each individual embryo to assure complete removal of the lipids.

Based on the above observations various groups have treated IVP swine embryos with trypsin to swell the zona pellucida [31], [32], or with high osmolality to shrink the embryo followed by centrifugation to separate the lipids. The high osmolality treated embryos were cultured to the blastocyst stage and subjected to cryopreservation in an OPS system. Live piglets resulted from both trypsin and high osmolality treated IVP embryos [18]. In another report IVP embryos were vitrified in a closed straw system and also produced live piglets [33]. The results presented here are somewhat different from those previously reported for IVP, in that 400 mOsm for 6 minutes of centrifugation was sufficient to completely separate the lipids and result in cryosurvival [18]. Similarly, the conditions for somatic cell nuclear transfer embryos was also different, e.g. the osmolality did not need to be as high for complete lipid separation [18].

Our original plan was to use the same media for in vivo-produced embryos that worked for generation of our preliminary data on our IVP embryos TCM199: [18]). Unfortunately, placing the in vivo-produced zygotes and 2-cell stage embryos in TCM199 for even a few min resulted in the embryos blocking at the 4-cell stage (unbpublished). This was quite surprising to us. While we knew that long term culture in TCM199 resulted in the embryos blocking [34], since the IVP embryos developed quite well after exposure to TCM199 we anticipated no problems with what we thought would be hardier in vivo-produced embryos. Nevertheless they blocked at the 4-cell stage. Thus we changed the base medium to TL-Hepes for centrifugation, and then the embryos developed to the blastocyst stage. The TL-Hepes was then used throughout the experiments.

There were significant differences due to the gilt from which the embryos were collected. Unfortunately since most of the collections were single animals on any given day, this variation is confounded with day. Similar differences due to gilt have been reported by us [35] and others [36] and indicate the tremendous variation due to different animals providing the embryos.

Treating in vivo-derived embryos with high osmolality and centrifugation to polarize intercellular lipid is compatible with development to blastocyst as seen with the development data. The rate of re-expansion of the blastocyst illustrates the survivability post-thawing of these embryos.

## Materials and Methods

All chemicals were purchased from Sigma (St. Louis, MO) unless otherwise indicated.

### Gilt Treatment and Embryo Collection

All experiments with animals were approved by the University of Missouri Institutional Animal Care and Use Committee (#6523). Surgery was performed under anesthesia, and analgesics were administered during the surgery. Pre-pubertal gilts were monitored for signs of estrous activity. When estrus was detected (day 0) the gilts were artificially inseminated 8 hours later. On Day 2 the gilt was anesthetized and oviducts flushed [37] with TL-Hepes [38] and embryos recovered. They were cultured in PZM3 (NaCl 108.0 mmol/L, KCl 10.0 mmol/L, KH_2_PO_4_ 0.35 mmol/L, MgSO_4_*7H_2_O 0.4 mmol/L, NaHCO_3_ 25.07 mmol/L, Na-pyruvate 0.2 mmol/L, Ca (Lactate)_2_ * 5 H_2_O 2.0 mmol/L, Glutamine 1.0 mmol/L, Hypotaurine 5.0 mmol/L, BME amino acid solution 20 ml/L, MEM amino acid solution 10 ml/L, Gentamicin 0.01 mg/mL, bovine serum albumin (BSA) 3 mg/mL, Osmolarity 288±2, pH 7.3±0.2) in 90% N_2_, 5% O_2_ and 5% CO_2_ at 38.5°C until treatment assignment. Embryos were randomly assigned to the various treatments (see Statistical Analysis below).

### High Osmolality Treatments

For the first aim zygotes and 2-cell stage embryos were collected and divided into 3 different osmolality treatment groups (300, 400 or 500 mOsm; achieved by adding sucrose) for 6 min and centrifuged for 0, 12, or 18 min. The durations were chosen to simulate what happens during the cryopreservation procedures as described above, i.e. they were all equilibrated for 6 min and then centrifuged for 6, 12 or 18 min. Then embryos were washed 3 times in PZM3 and cultured for 6 days in PZM3. This experiment determined how sensitive the in vivo-produced embryos are to high osmolality treatments induced by sucrose.

Based on the results from the first aim the following treatments were chosen for the second aim, for preparation prior to vitrification: 1) 300 mOsm centrifuged for 6 min, 2) 400 mOsm centrifuged for 12 min, 3) 500 mOsm centrifuged for 12 min, or 4) 500 mOsm centrifuged for 18 min. The embryos were cultured to the blastocyst stage and vitrified.

### Vitrification

Vitrification of embryos was carried out by using the OPS method. The OPS straws were purchased from LEC Instruments P/L and Minitube. All solutions used during vitrification were prepared with holding medium (Oocyte manipulation medium with 20% fetal calf serum instead of 3 mg/mL BSA). Blastocysts were placed in equilibration solution (10% ethylene glycol, 10% dimethyl sulfoxide [DMSO]) for 2 min, followed by exposure to vitrification solution (20% ethylene glycol, 20% DMSO). Embryos were loaded into an OPS and immediately plunged into liquid nitrogen. All processes before plunging into nitrogen were conducted on a 37°C warm stage. The time from exposure to the vitrification solution to plunging was 25–30 sec.

For embryo warming, cryopreserved embryos were warmed by removing the straw from liquid nitrogen for a few seconds and immersing the end of the OPS into 0.3 M sucrose for 6 min at 37°C, then transferring them to 0.2 M sucrose for 6 min, and then holding medium for 6 min. The warmed embryos were cultured for 18 hours in PZM3 with 10% FBS to check re-expansion, and nuclear number.

### Statistical Analysis

Embryos from a single gilt were selected at random and assigned to the treatments. Since often the number of embryos from a single gilt did not match the number of treatments additional embryos were assigned to other treatments in order. So, for example, if there are seven treatments and 10 embryos are collected, then seven treatments received 1 embryo and three treatments received 2 embryos. The next gilt provided embryos first to the last four treatments that only had a single embryo, and then the remainder were distributed to the other treatments. In contrast, the same strategy was applied if fewer than seven embryos were collected. It should be noted that in some cases 2- and 4-cell stage embryos were collected. In this case the 4-cell stage embryos were not used. Similarly, some oocytes were unfertilized; these also were not used. This strategy permitted a robust analysis of the treatment effects as well as providing an opportunity to detect gilt effects. All data were analyzed by using the PROC GLM commands in SAS 9.1 (Cary, NC, USA). The main effects of the number of nuclei in the blastocysts were tested as appropriate in each experiment. Gilt was also included in the model to remove variation due to different animals. Percentage data was arcsine transformed prior to analysis. Differences between treatment groups (time by osmolality) were assessed via post-hoc pairwise comparisons of least squares mean values. Significance was assigned at P-values <0.05.

## References

[1] Polge C, Wilmut I, Rowson LEA (1977) The low temperature preservation of cow, sheep, and pig embryos. Cryobiology 11: 560.

[2] Wilmut I (1972) The low temperature preservation of mammalina embryos. Journal of Reproduction and Fertility 31: 513–514. 4120076 10.1530/jrf.0.0310513

[3] Dobrinsky JR (1996) Cellular Approach to Cryopreservation of Embryos. Theriogenology 45: 17–26.

[4] Nagashima H, Kashiwazaki N, Ashman RJ, Grupen CG, Seamark RF, et al (1994) Removal of Cytoplasmic Lipid Enhances the Tolerance of Porcine Embryos to Chilling. Biology of Reproduction 51: 618–622. 7819441 10.1095/biolreprod51.4.618

[5] Beebe LFS, Cameron RDA, Blackshaw AW, Keates HL (2005) Changes to porcine blastocyst vitrification methods and improved litter size after transfer. Theriogenology 64: 879–890. 16054493 10.1016/j.theriogenology.2004.12.014

[6] Dobrinsky JR, Pursel VG, Long CR, Johnson LA (2000) Birth of piglets after transfer of embryos cryopreserved by cytoskeletal stabilization and vitrification. Biology of Reproduction 62: 564–570. 10684796 10.1095/biolreprod62.3.564

[7] Berthelot F, Martinat-Botte F, Perreau C, Terqui M (2001) Birth of piglets after OPS vitrification and transfer of compacted morula stage embryos with intact zona pellucida. Reproduction, Nutrition, Development 41: 267–272. 10.1051/rnd:2001129 11592724

[8] Beebe LFS, Cameron RDA, Blackshaw AW, Higgins A, Nottle MB (2002) Piglets born from centrifuged and vitrified early and peri-hatching blastocysts. Theriogenology 57: 2155–2165. 12141566 10.1016/s0093-691x(01)00720-8

[9] Misumi K, Suzuki M, Sato S, Saito N (2003) Successful production of piglets derived from vitrified morulae and early blastocysts using a microdroplet method. Theriogenology 60: 253–260. 12749938 10.1016/s0093-691x(02)01364-x

[10] Men HS, Agca Y, Riley LK, Critser JK (2006) Improved survival of vitrified porcine embryos after partial delipation through chemically stimulated lipolysis and inhibition of apoptosis. Theriogenology 66: 2008–2016. 16870242 10.1016/j.theriogenology.2006.05.018

[11] Fujino Y, Kikuchi K, Nakamura Y, Kobayashi H, Yonemura I, et al (2006) Batchwise assessment of porcine embryos for cryotolerance. Theriogenology 67: 413–422. 16987547 10.1016/j.theriogenology.2006.08.008

[12] Nagashima H, Kashiwazaki N, Ashman RJ, Grupen CG, Nottle MB (1995) Cryopreservation of porcine embryos. Nature 374: 416. 7700349 10.1038/374416a0

[13] Nagashima H, Cameron RD, Kuwayama M, Young M, Beebe L, et al (1999) Survival of porcine delipated oocytes and embryos after cryopreservation by freezing or vitrification. Journal of Reproduction and Development 45: 167–176.

[14] Li R, Lai L, Wax D, Hao Y, Murphy CN, et al (2006) Cloned transgenic swine via in vitro production and cryopreservation. Biology of Reproduction 75: 226–230. 16672718 10.1095/biolreprod.106.052514

[15] Lai LX, Kang JX, Li RF, Wang JD, Witt WT, et al (2006) Generation of cloned transgenic pigs rich in omega-3 fatty acids. Nature Biotechnology 24: 435–436. 10.1038/nbt1198 PMC2976610 16565727

[16] Wrathall AE, Sutmoller P (1998) Potential of embryo transfer to control transmission of disease. In: Stringfellow DA, Seidel SM, editors. Manual of the International Embryo Transfer Society. 3rd ed. Savoy, IL: International Embryo Transfer Society. 17–44.

[17] Stringfellow DA (1998) Recommendations for the sanitary handling of in-vivo-derived embryos. In: Stringfellow DA, Seidel SM, editors. Manual of the International Embryo Transfer Society. 3rd ed. Savoy, IL: International Embryo Transfer Society. 79–84.

[18] Li R, Murphy CN, Spate L, Wax D, Isom C, et al (2009) Production of piglets after cryopreservation of embryos using a centrifugation-based method for delipation without micromanipulation. Biol Reprod 80: 563–571. 19038857 10.1095/biolreprod.108.073387 PMC2755258

[19] Mullen SF, Rosenbaum M, Critser JK (2007) The effect of osmotic stress on the cell volume, metaphase II spindle and developmental potential of in vitro matured porcine oocytes. Cryobiology 54: 281–289. 17485076 10.1016/j.cryobiol.2007.03.005 PMC1989776

[20] Men HS, Agca Y, Mullen SF, Critser ES, Critser JK (2005) Osmotic tolerance of in vitro produced porcine blastocysts assessed by their morphological integrity and cellular actin filament organization. Cryobiology 51: 119–129. 16024011 10.1016/j.cryobiol.2005.05.005

[21] Yoshioka K, Suzuki C, Tanaka A, Anas IMK, Iwamura S (2002) Birth of piglets derived from porcine zygotes cultured in a chemically defined medium. Biology of Reproduction 66: 112–119. 11751272 10.1095/biolreprod66.1.112

[22] Norberg HS (1973) Ultrastructural aspects of the preattached pig embryo: cleavage and early blastocyst stage. Z Anat Entwicklungsgesch 143: 95–114. 4801840 10.1007/BF00519913

[23] Kikuchi K, Ekwall H, Tienthai P, Kawai Y, Noguchi J, et al (2002) Morphological features of lipid droplet transition during porcine oocyte fertilisation and early embryonic development to blastocyst in vivo and in vitro. Zygote 10: 355–366. 12463532 10.1017/s0967199402004100

[24] Nagashima H, Kato Y, Yamakawa H, Matsumoto T, Ogwa S (1989) Changes in freezing tolerance of pig blastocysts in peri-hatching stages. Jpn J Animal Reproduction 35: 130–134.

[25] Nagashima H, Yamakawa H, Niemann H (1992) Freezability of porcine blastocysts at different peri-hatching stages. Theriogenology 37: 839–850. 16727083 10.1016/0093-691x(92)90045-s

[26] Li R, Hosoe M, Shioya Y, Bou S (2002) The preliminary research on freezing viability of bovine in vitro fertilized embryos. Chinese J Scientia Agricultura Sinica 35: 1125–1129.

[27] McEvoy TG, Coull GD, Broadbent PJ, Hutchinson JSM, Speake BK (2000) Fatty acid composition of lipids in immature cattle, pig and sheep oocytes with intact zona pellucida. Journal of Reproduction & Fertility 118: 163–170. 10793638

[28] Nagashima H, Hiruma K, Saito H, Tomii R, Ueno S, et al (2007) Production of live piglets following cryopreservation of embryos derived from in vitro-matured oocytes. Biology of Reproduction 76: 900–905. 17267701 10.1095/biolreprod.106.052779

[29] Dobrinsky JR (2002) Advancements in cryopreservation of domestic animal embryos. Theriogenology 57: 285–302. 11775976 10.1016/s0093-691x(01)00672-0

[30] Cameron RDA, Beebe LFS, Blackshaw AW, Keates HL (2004) Farrowing rates and litter size following transfer of vitrified porcine embryos into a commercial swine herd. Theriogenology 61: 1533–1543. 15036983 10.1016/j.theriogenology.2003.09.003

[31] Esaki R, Ueda H, Kurome M, Hirakawa K, Tomii R, et al (2004) Cryopreservation of porcine embryos derived from in vitro-matured oocytes. Biology of Reproduction 71: 432–437. 15044264 10.1095/biolreprod.103.026542

[32] Du Y, Zhang Y, Li J, Kragh PM, Kuwayama M, et al (2007) Simplified cryopreservation of porcine cloned blastocysts. Cryobiology 54: 181–187. 17359960 10.1016/j.cryobiol.2007.01.001

[33] Men H, Zhao C, Si W, Murphy CN, Spate L, et al (2011) Birth of piglets from in vitro-produced, zona-intact porcine embryos vitrified in a closed system. Theriogenology 76: 280–289. 21458047 10.1016/j.theriogenology.2011.02.004 PMC3115500

[34] Hagen DR, Prather RS, Sims MM, First NL (1991) Development of one-cell porcine embryos to the blastocyst stage in simple media. J Anim Sci 69: 1147–1150. 2061246 10.2527/1991.6931147x

[35] Prather RS, Boice ML, Gibson J, Hoffman KE, Parry TW (1995) In vitro development of embryos from Sinclair miniature pigs: a preliminary report. Theriogenology 43: 1001–1007. 16727687 10.1016/0093-691x(95)00064-f

[36] Petters RM, Johnson BH, Reed ML, Archibong AE (1990) Glucose, glutamine and inorganic phosphate in early development of the pig embryo in vitro. J Reprod Fertil 89: 269–275. 2374120 10.1530/jrf.0.0890269

[37] Machaty Z, Day BN, Prather RS (1998) Development of Early Porcine Embryos in Vitro and in Vivo. Biology of Reproduction 59: 451–455. 9687321 10.1095/biolreprod59.2.451

[38] Im G-S, Lai L, Liu Z, Hao Y, Prather RS (2004) In vitro development of preimplantation porcine nuclear transfer embryos cultured in different media and gas atmospheres. Theriogenology 61: 1125–1135. 15037000 10.1016/j.theriogenology.2003.06.006

